# A Point-of-Care Nucleic Acid Quantification Method by Counting Light Spots Formed by LAMP Amplicons on a Paper Membrane

**DOI:** 10.3390/bios14030139

**Published:** 2024-03-10

**Authors:** Yanju Chen, Yuanyuan Zhu, Cheng Peng, Xiaofu Wang, Jian Wu, Huan Chen, Junfeng Xu

**Affiliations:** 1 ZJU-Hangzhou Global Scientific and Technological Innovation Center, College of Biosystems Engineering and Food Science, Zhejiang University, Hangzhou 310058, China; 2 Key Laboratory of Traceability for Agricultural Genetically Modified Organisms, Ministry of Agriculture and Rural Affairs, Zhejiang Academy of Agricultural Sciences, Hangzhou 310021, China; 3 Hangzhou Digital-Micro Biotech Co., Ltd., Hangzhou 311215, China; chenhuan7809@gmail.com

**Keywords:** nucleic acid quantification, digital detection, paper membrane, probe-based LAMP

## Abstract

Nucleic acid quantification, allowing us to accurately know the copy number of target nucleic acids, is significant for diagnosis, food safety, agricultural production, and environmental protection. However, current digital quantification methods require expensive instruments or complicated microfluidic chips, making it difficult to popularize in the point-of-care detection. Paper is an inexpensive and readily available material. In this study, we propose a simple and cost-effective paper membrane-based digital loop-mediated isothermal amplification (LAMP) method for nucleic acid quantification. In the presence of DNA fluorescence dyes, the high background signals will cover up the amplicons-formed bright spots. To reduce the background fluorescence signals, a quencher-fluorophore duplex was introduced in LAMP primers to replace non-specific fluorescence dyes. After that, the amplicons-formed spots on the paper membrane can be observed; thus, the target DNA can be quantified by counting the spots. Take *Vibrio parahaemolyticus* DNA detection as an instance, a good linear relationship is obtained between the light spots and the copy numbers of DNA. The paper membrane-based digital LAMP detection can detect 100 copies target DNA per reaction within 30 min. Overall, the proposed nucleic acid quantification method has the advantages of a simple workflow, short sample-in and answer-out time, low cost, and high signal-to-noise, which is promising for application in resourced limited areas.

## 1. Introduction

Nucleic acid amplification-based detection is important for medical diagnosis, food safety, agricultural production, and environmental monitoring [1]. Quantitative nucleic acid analysis provides accurate information about the concentration of target nucleic acids. For example, the virus loads are related to the severity of illness and clinical symptoms, and the concentration of bacteria influences the freshness of foods [2,3].

Polymerase chain reaction (PCR) is the gold standard of molecular detection. Quantitative PCR (qPCR) is widely used for quantifying target nucleic acids. However, it requires a standard sample to establish calibration curves [4]. Sometimes, it is difficult to acquire standards of some nucleic acid samples. Furthermore, the nucleic acid standards, particularly RNA, are prone to degradation over time, causing accuracy and reproducibility deviations [5,6]. The expensive real-time fluorescence thermal cyclers also increase the cost of qPCR-based quantification detection. The development of digital PCR (dPCR) has revolutionized qPCR by allowing for the direct quantification of targets without calibration curves [7]. The commercial platforms such as QuantStudio 3D Digital PCR, BioRad QX200, and Qiagen digital PCR system, promote the practical application of dPCR [8]. However, these digital platforms require much more expensive instrumentations and reagents than qPCR. The operation steps are also complex and require trained staff. For example, when performing dPCR using the BioRad QX200 system, it is necessary to use the corresponding reagents produced by BioRad company, increasing the cost of detection. In addition, the entire steps take more than 3 h, including PCR mixture preparation, droplet production, PCR, and droplet signal detection [9,10]. In summary, either the semi-quantitative qPCR or the absolute quantitative dPCR method requires expensive instruments and trained staff, which makes it difficult to further popularize nucleic acid quantitative detection.

Compared with PCR, isothermal amplification technologies are more suitable for point-of-care detection due to the constant reaction temperature, especially in resource-limited areas. The constant temperature reaction allows the amplification process to be completed in the heat block, reducing the requirements for temperature control instruments. The reported isothermal amplification technologies include strand displacement amplification (SDA), helicase-dependent amplification (HDA), nucleic acid sequence-based amplification (NASBA), recombinase polymerase amplification (RPA), rolling circle amplification (RCA), and loop-mediated isothermal amplification (LAMP) [11]. Among various isothermal amplification reactions, RPA and LAMP reactions have shorter reaction time and higher amplification efficiency. Several amplification reactions require the participation of two or more enzymes. The primer design of the LAMP reaction is complex, requiring 4–6 primers in the reaction system. In order to solve this problem, researchers have developed the software or the website for designing LAMP primers, which has greatly reduced the difficulty of designing LAMP primers, making the LAMP reaction more widely used [12]. In general, LAMP amplification, as one of the most widely used isothermal amplification methods, has the following advantages: (1) The reaction temperature is around 65 °C and does not require a pre-heating step. (2) The reaction is highly resistant to interference. (3) The reaction process requires only one enzyme [13].

Based on the above advantages, many LAMP-based detection methods were established and could be classified into real-time and end-point detection methods. For the real-time LAMP methods, fluorescent dyes or probes are added into the reaction system, allowing the amplification process to be monitored by the instrument. The real-time detection method has high requirements for the detector and is not suitable for the point-of-care detection. The endpoint LAMP detection methods include gel electrophoresis, turbidity, metal indicator, test strip, etc. The gel electrophoresis method decides the LAMP amplification products based on the characteristic bands. The turbidity method determines whether the amplification proceeds by the white precipitate of magnesium pyrophosphate produced during the LAMP amplification process. The metal indicator method determines whether the reaction proceeds by the changes in magnesium ions in the reaction system before and after LAMP amplification. The test strip detection method modifies two primers in the LAMP system so that the amplified product can be combined with colloidal gold to develop color at the test line [14]. However, for the endpoint LAMP detection methods, it is difficult to quantify the target nucleic acids.

The essence of digital detection is “partition, amplification, detection”. Microfluidic platforms can perform quantitative nucleic acid analysis by separating reaction mixture in small-volume microwells or the generation of water-in-oil droplets on a chip [15]. The water-in-oil droplets can be produced by a “T-shaped” junction, a “cross” flow focusing junction, centrifugation, or step emulsification [16,17,18]. For microwell-based chips, small containers are patterned by microfabrication on glass, silicon, polydimethylsiloxane (PDMS), or other polymers [19,20,21,22]. Although these microfluidics-based digital platforms have lower costs than commercial instruments, they require elaborate chip design and complex fabrication steps, which complicates the detection process and is unfriendly to remote areas.

To establish point-of-care LAMP-based detection methods, Huang et al. developed a gel-based digital LAMP method by restricting the nucleic acids in the hydrogel matrix [23]. Zhu et al. applied the disposable needle and microcentrifuge tube to generate hydrogel beads for digital LAMP [24]. For the above methods, the LAMP solution should be mixed with a gel solution, and then the LAMP mixture can be partitioned. Therefore, the gel or hydrogel beads need to be prepared and used immediately. Lin et al. proposed a commercial track-etched polycarbonate (PTCE) membrane-assisted digital method by dropping and partitioning the LAMP mixture into the cylindrical pores of the PTCE membrane [25]. The residual liquids on the surface of the membrane were removed by peeling off the PDMS film, and then a layer of mineral oil was dropped to cover the pores to prevent evaporation. The commercial membrane is available and the cost is low. However, in this study, the polyvinylpyrrolidone (PVP) coating on the surface needed to be removed before use.

**Figure 1:**
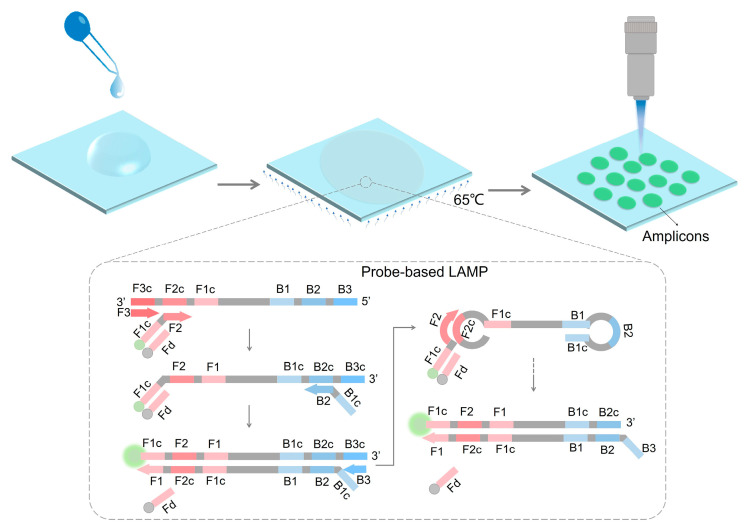
The diagram of probe-based LAMP reaction on a paper membrane for nucleic acid quantification.

Paper is a cheap material that can replace polymer microfluidic materials such as glass, PDMS, and plastics. It has been reported that paper microfluidics can integrate nucleic acid extraction, amplification, and detection processes [26,27]. Previously, it is reported that some nucleic acid amplification processes can be conducted on the paper membranes. Some people established paper-based LAMP colorimetric methods [28]. In this study, we found that the porous structure of the paper membrane enables the amplification solution to be absorbed, thereby forming small, compartmentalized reaction chambers naturally. Based on this, we propose a digital loop-mediated isothermal amplification (LAMP) platform for quantifying target nucleic acid by counting “light spots” on the paper membrane. DNA dye is commonly used for the detection of LAMP amplicons. We find that DNA dyes lead to high background signals on the paper membrane. Therefore, we designed a fluorescence and quencher-modified probe to replace non-specific DNA dye in the LAMP reaction. In this way, the background signals can be significantly reduced by replacing the DNA dye with the probes. LAMP amplicons will form discrete light spots on the membrane after probe-based LAMP amplification (Figure 1). The number of light spots has a good linear relationship with the copy number of the target DNA (100–1000 copies). The proposed detection method, based on a paper membrane, can achieve nucleic acid quantification within 30 min. This nucleic acid quantification platform has the advantages of simplicity, low cost, short sample-in-answer-out time, and high signal-to-noise.

## 2. Materials and Methods

### 2.1. Material

The paper membranes commonly used in the laboratory were selected for LAMP amplification, including Nylon membrane (GNWP02500), Mixed Cellulose Esters (MCE, VSWP02500), and polyethersulfone (PES, GPWP04700), which were purchased from EMD Millipore (Billerica, MA, USA). The *V. parahaemolyticus* O3:K6 was provided by Lin’an Center for Disease Control and Prevention (Hangzhou, China). Bacterial DNA was extracted by a commercial TIANamp genomic DNA kit (TIANGEN Biotech Co., Ltd., Beijing, China). To know the accurate copy number, the extracted sample was absolutely quantified by digital PCR using the QX200 Bio-Rad droplet digital PCR system.

### 2.2. Membrane Characterization and Water Absorbency Testing

The morphologies of the membranes were visualized with a field emission scanning electron microscope (Hitachi SU-8010, Tokyo, Japan). SEM uses electrons instead of light to form an image. SEM can provide nanoscale information of the sample. The traditional SEM requires the sample to be electronically conductive because SEM imaging is produced by detecting the signals of the electrons. If the sample is non-conductive or has poor conductivity, the surface of the sample can act as an electron trap. Excess electrons will accumulate on the surface of the sample and interfere with the transmission of the signals of the electrons, affecting the imaging process. In this paper, the membranes are non-conductive materials. Therefore, the membrane was put into a sputter coater (Hitachi, Tokyo, Japan) to sputter a gold film on the surface of the membrane. Each paper membrane was cut into 2 mm by 5 mm rectangles and the surface was coated with gold before imaging. The water absorbency was tested by adding different volumes of water to the 1.4 cm by 1.4 cm squares of the paper membrane. The maximum volume of water that can be absorbed per unit area of the paper membrane was defined as the water absorbency (µL/cm^2^).

### 2.3. LAMP Reaction System

The LAMP primers were designed by our group using PrimerExplore V4 software. The DNA sequences were commercially synthesized by Sangon (Shanghai, China). The sequence information is listed in Table S1. The LAMP reaction system contained 1× isothermal amplification buffer, 2 mM MgSO_4_ (New England Biolabs Inc., Ipswich, MA, USA), 16 U Bst 2.0 WarmStart DNA polymerase (New England Biolabs Inc., Ipswich, MA, USA), 0.8 M Betainee (Sigma-Aldrich Co. LLC., St. Louis, MO, USA), 1.4 mM dNTP mixtures, 1.6 µM FIP, 1.6 µM BIP, 0.2 µM F3, 0.2 µM B3, 0.4 µM LB, 0.4 µM LF, 2 µL target DNA. For dye-based LAMP, 2 µM SYTO 9 stains (Thermo Fisher Scientific Inc., Waltham, MA, USA) were added to the reaction system. For probe-based LAMP, the equal concentration of F-FIP and Q-Fd mixtures were heated at 95 °C for 10 min and then annealed to room temperature to form the FIP/Fd probe. The total concentration of the FIP and FIP/Fd probe remains 0.4 µM. For comparison of the dye-based and probe-based LAMP assay, 50% FIP/Fd probes were used in the probe-based LAMP. The real-time LAMP detection signals were collected by QuantStudio 3 Real-Time PCR System (Thermo Fisher Scientific Inc., Waltham, MA, USA).

### 2.4. LAMP Reaction on the Paper Membranes

For the LAMP reaction on the membrane, the volume of amplification solution added to the membrane was slightly less than the maximum water absorbency of the membranes. The paper membrane was cut into 1.4 cm by 1.4 cm squares and the LAMP mixture was pipetted on it. After the LAMP solution was absorbed by the paper membrane, the paper membrane was placed on a glass slide (5.5 cm × 5.5 cm × 0.55 cm) and sealed by the PCR sealing membrane (4 cm × 4 cm) (Sangon, Shanghai, China). The glass sheet was heated at 65 °C on the heat plate (Chemat Technology, Shanghai, China) for the LAMP reaction. The temperature of the upper surface of the glass slide and the heat plate were tested by a thermocouple.

### 2.5. Feasibility of LAMP Reaction on the Paper Membranes

To test the feasibility of the LAMP reaction on the paper membranes, after 30 min LAMP reaction, the PCR sealing membrane attached to the glass slide was uncovered and the center of the paper membrane was cut off into a disc with a diameter of 2 mm. The disc was put into CRISPR/Cas12a solution for further detection. Here, the LAMP reaction system did not contain SYTO 9 dyes or probes. The 20 µL CRISPR/Cas12a detection system contained 150 nM Cas12a protein (New England Biolabs Inc., Ipswich, MA, USA), 600 nM crRNA, 2 µM ssDNA probes, 1× NEB buffer 2.1 (New England Biolabs Inc., Ipswich, MA, USA), and 10 U recombinant RNase inhibitor (Takara Biomedical Technology Co., Ltd., Beijing, China). CRISPR detection signals were collected by the QuantStudio 3 Real-Time PCR System (Thermo Fisher Scientific Inc., Waltham, MA, USA).

### 2.6. Light Spot Observation on the Paper Membrane

After 30 min LAMP reaction on the heat plate, the glass side was imaged by a Nikon SMZ18 fluorescence microscope with 0.5× objective (Nikon, Japan). The exposure time was 900 ms. The entire field of view of the paper membrane can be captured through this microscope. For the fluorescence signals on paper membranes, the red, green, and blue (RGB) values of the fluorescence spots were extracted by MATLAB 2022b software. The light spots on the paper membranes were counted by Image J 1.54f after setting the threshold.

## 3. Results and Discussion

### 3.1. Characterization of Paper Membrane

**Figure 2:**
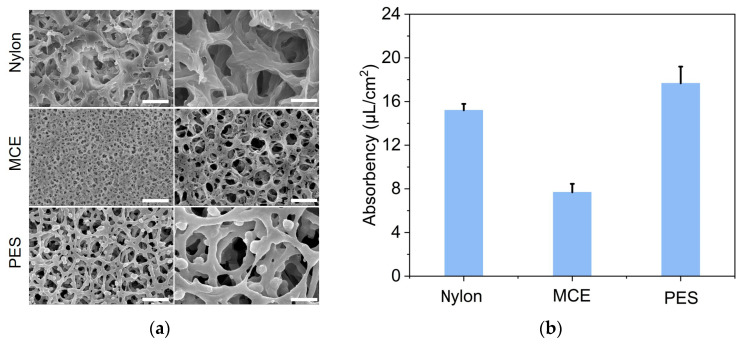
(**a**) Scanning electron micrograph of Nylon, MCE, and PES. The scale bars on the left and right represent 1 µm and 300 nm, respectively. (**b**) The water absorbency of the three paper membranes.

To realize LAMP-based quantification on paper membranes, firstly, we selected three common commercial membranes (Nylon, MCE, PES) in laboratories. The SEM diagrams showed the surface morphology of these membranes (Figure 2a). All three membranes had high porosity and tortuous pores. The pore size of the MCE membrane was found to be smaller than the Nylon and PES.

It might be a little difficult for the absorption of the solution into the MCE membrane. To achieve the on-paper LAMP reaction, it is necessary to ensure that the LAMP mixture can be absorbed by the paper membrane. We tested the water absorption capacity of these three paper membranes. According to the test results in Figure 2b, the Nylon and PES membranes exhibited a higher water absorption than the MCE, which indicated that water could diffuse smoothly on the Nylon and PES membranes.

### 3.2. Feasibility of LAMP Amplification on the Paper Membrane

In this study, one of the most common foodborne pathogens *Vibrio parahemolyticus* was used as the detection target. According to WHO, nearly 600 million people worldwide (almost 1 in 10 people) become ill each year due to contaminated food. Foodborne diseases have become a worldwide public health problem. Foodborne pathogens are currently one of the main factors that leads to foodborne diseases. *Vibrio parahaemolyticus* and *Salmonella* are the two main foodborne pathogenic bacteria. *Vibrio parahaemolyticus* has a high tolerance and strong survivability in high-salt environments. Seafood, especially fish, shrimp, and shellfish, are the main source of *Vibrio parahaemolyticus*. China, especially the southern coastal areas, has a high infection rate with *Vibrio parahaemolyticus* [29]. Therefore, there is an urgent need to establish a fast, sensitive, and reliable method for *Vibrio parahaemolyticus* detection. Apart from *Vibrio parahaemolyticus*, the established method can also be used for the detection of other microorganisms by designing the primers and probes according to the specific nucleic acid sequence of the target microorganisms. In this paper, *Vibrio parahaemolyticus* was used to verify the feasibility of the established method.

**Figure 3:**
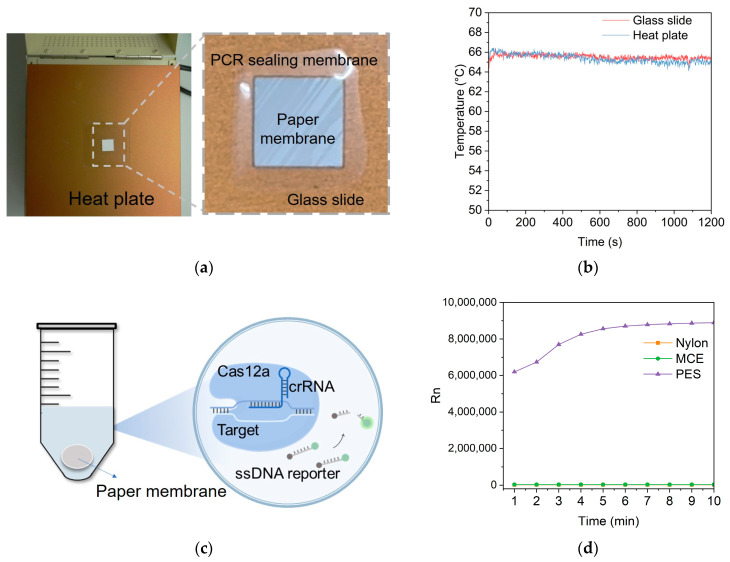
(**a**) The PES membrane was placed on the glass slide and covered with PCR sealing tape. (**b**) The temperature of the heat plate and the glass slide when setting the temperature at 65 °C. (**c**) The CRISPR/Cas12a-based method for detection of LAMP amplicons on the paper membrane. (**d**) The results of CRISPR/Cas12a for detection of amplicons on paper membranes. Rn is normalized fluorescence signals.

The Nylon, MCE, and PES paper membranes were cut into 1.4 cm by 1.4 cm squares. The LAMP amplification mixtures were added to the three squares, respectively. Following that, these squares were put on the glass slides and then covered by PCR sealing membranes. The glass slides were put on the heat plate for the LAMP reaction (Figure 3a). The temperature of the upper surface of the glass slide and the heat plate was tested by a thermocouple when the temperature was set at 65 °C. The results showed that the temperature on the heat plate and the glass slide could maintain 65 °C and fluctuate within 1 °C, providing a stable incubation temperature for the LAMP reaction on paper membranes (Figure 3b). To investigate the feasibility of the LAMP reaction on the paper membranes, the CRISPR/Cas12a system was applied to detect the LAMP amplicons on the paper membranes (Figure 3c). CRISPR/Cas12a is a novel nucleic acid detection tool with high specificity. It can be used to detect LAMP amplicons. CRISPR/Cas12a system, composed of crRNA, Cas12a protein, and the fluorophore and quencher-modified ssDNA reporters, is a novel nucleic acid detection tool. Once the LAMP amplicons are recognized by the Cas12a-crRNA complex, it will nonspecifically cut the ssDNA reporter and produce fluorescence signals caused by the division of fluorophore and quencher. According to the detection results in Figure 3d, only the PES membrane in the CRISPR/Cas12a solution produced fluorescence signals, indicating that the LAMP reaction could be successfully performed on the PES membrane. The experimental results align with the previous study. The specific mechanism of how the LAMP reaction can be conducted inside the matrix of the PES membrane needs to be further explored. Based on our research and the relevant literature, this may be related to the water absorption capability of membranes and non-specific protein absorption. It is necessary to contain the LAMP solution in the pores inside the membrane. The LAMP reaction is conducted at 65 °C. If the reaction solution cannot be locked inside the membrane, the solution in the membrane will evaporate, leading to the failure of the LAMP reaction. The PES membrane shows a higher water absorption capability than the other two membranes. In addition, the LAMP amplification process requires the participation of DNA polymerase. It is reported that the PES membrane shows low non-specific protein absorption. These two points may be the reason why LAMP can be successfully carried out on PES [30].

### 3.3. LAMP Reaction on the PES Membrane

**Figure 4:**
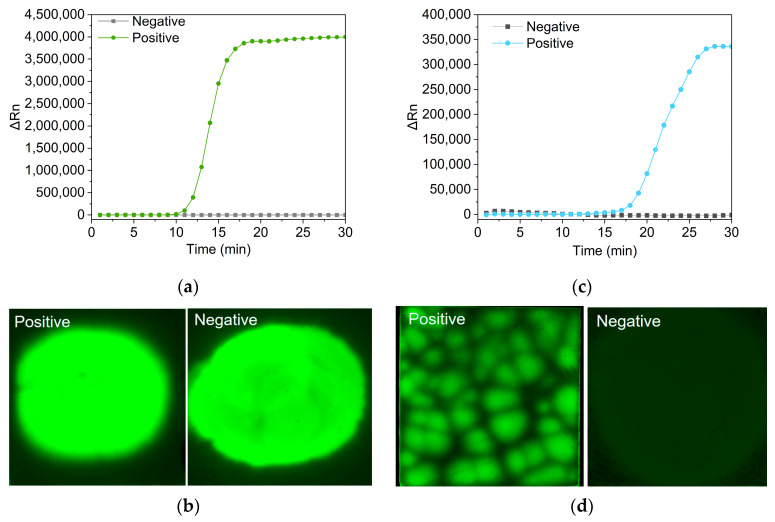
(**a**) The real-time curve of intercalating fluorescence dye-based LAMP. (**b**) The photographs of the PES membrane after dye-based LAMP. (**c**) The real-time curve of FIP/Fd probe-based LAMP. (**d**) The photographs of the PES membrane after probe-based LAMP. Rn is normalized fluorescence signals.

LAMP is a well-known isothermal amplification method for its high sensitivity and specificity, which is realized by the utilization of up to six primers. A common method for the detection of amplicons is to add intercalating fluorescence dyes in the LAMP reaction system. It was reported that the SYBR green and EVAGreen dyes had an inhibitory effect on the efficiency of the real-time LAMP assay. SYTO 9 dye represented no inhibitory effect [31]. Therefore, in this study, SYTO 9 dye was added to the LAMP reaction and a real-time curve could be gained by the real-time PCR machine (Figure 4a). A LAMP reaction solution containing SYTO 9 dye was also added onto the PES membrane for on-paper LAMP. However, it was found that the positive and the negative samples both had strong fluorescence signals on the PES membranes, making it difficult to distinguish (Figure 4b). To reduce the background signals, we introduced a quencher-fluorophore duplex region in LAMP primers, allowing real-time fluorescence detection by strand displacement of the quencher-modified sequence in the process of probe-based amplification (Figure 4c). The probe-based amplification solution was also pipetted onto the PES membranes for LAMP amplification. It was found that positive and negative samples had distinct fluorescence differences (Figure 4d). The probe-based LAMP reduced the background signal on the PES membranes. More importantly, there were isolated light spots on the PES membrane of the positive sample, which indicated its potential for nucleic acid quantification.

### 3.4. Optimization of Probe-Based LAMP Reaction

**Figure 5:**
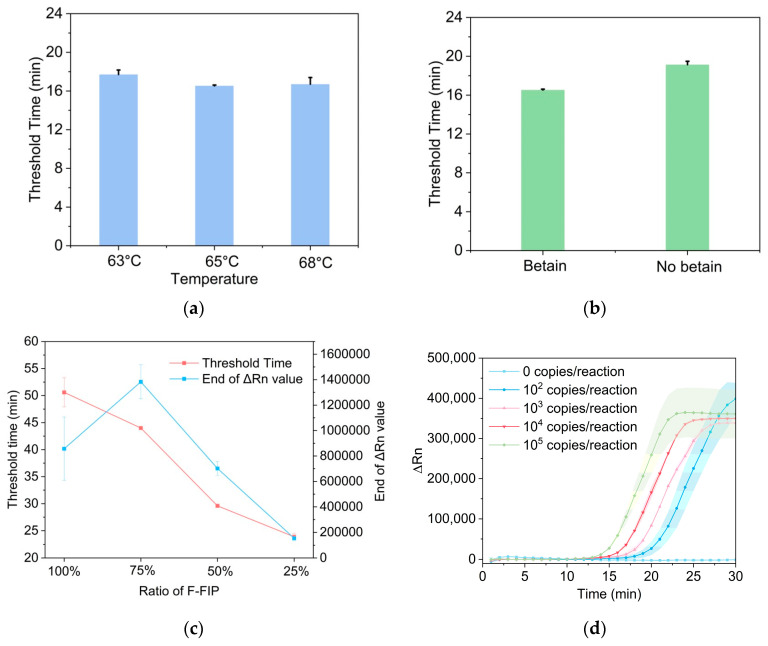
(**a**) Optimization of reaction temperature of the probe-based LAMP. (**b**) The influence of betaine on the probe-based LAMP. (**c**) The influence of the different ratios of F-FIP to total FIP on the threshold time and the end-point fluorescence of probe-based LAMP. (**d**) The sensitivity of the probe-based LAMP was evaluated by the detection of a ten-fold diluent of *Vibrio parahemolyticus* DNA. Rn is normalized fluorescence signals.

The probe-based LAMP is more suitable for on-paper detection due to its lower background signals and higher signal-to-noise. The probe used in the probe-based LAMP is composed of a 5′-fluorophore-modified FIP and a 3′-quencher-modified-Fd. In this paper, the fluorophore and quencher groups are FAM and BHQ1, respectively. The FIP and Fd sequences will hybridize and form a duplex probe after the denaturation-annealing step. The fluorescence signals of the duplex probe will be quenched due to the proximity of the fluorophore and quencher. Once the target nucleic acid is present, the LAMP reaction will proceed and the Fd sequence will be displaced. Therefore, there will be an increasing fluorescence signal. Compared with the dye-based LAMP methods, the probe-based method has higher specificity but a slower reaction rate [32]. Here, we optimized the reaction conditions to accelerate the probe-based LAMP reaction. The threshold time was applied as the evaluation parameter and a smaller threshold time value indicated a faster reaction. We compared the reaction rate of the probe-based LAMP at different temperatures, and the results showed that LAMP could be performed at around 65 °C (Figure 5a). There are no particularly precise requirements for temperature, which is also suitable for point-of-care detection. It was reported that the introduction of betaine into the LAMP reaction could reduce base stacking and increase the reaction speed, especially in the sequences with a high GC content [33]. We found that the inclusion of betaine could lightly accelerate the probe-based LAMP reaction (Figure 5b). The proportion of FIP/Fd probes in the probe-based LAMP was an important factor for reaction speed. According to the detection results in Figure 5c, increasing the ratio of F-FIP in the reaction system delayed the threshold time of the probe-based LAMP, which meant that the reaction was slowed down. The end-point fluorescence value also increased as the concentration of F-FIP improved. The fluorescence value of 100% F-FIP was lower than 75% F-FIP due to the limitations of reaction time. 50% F-FIP was finally selected for the next probe-based LAMP after a comprehensive consideration of the reaction speed and fluorescence intensity. A series of concentrations of extracted *Vibrio parahemolyticus* DNA was detected by the probe-based LAMP at the optimal condition. The results showed that the probe-based LAMP could detect as low as 100 copies of *Vibrio parahemolyticus* DNA per reaction within 30 min (Figure 5d).

### 3.5. Vibrio Parahemolyticus DNA Quantification on the PES Membrane

**Figure 6:**
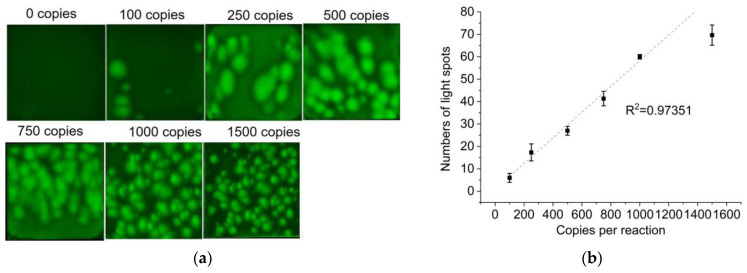
(**a**) Representative fluorescence images of probe-based LAMP products on the PES membrane taken by a microscope. (**b**) The correlation relationship between the number of light spots and the copy number of target DNA.

The extracted DNA was diluted into different concentrations with a TE buffer, and then detected by the established method. The probe-based LAMP mixture, containing different copies of *Vibrio parahemolyticus* DNA, was dropped onto the PES membranes and incubated on the established platform. The PES membranes were imaged by a microscope after a 30 min LAMP reaction. According to the detection results, higher concentrations of target DNA formed more light spots on the PES membrane. The light spots on the PES membrane appeared smaller with the higher concentration of target DNA. When the concentration of target DNA was 100–1000 copies, discrete light spots were displayed on the PES membranes (Figure 6a). By counting the light spots on the PES membrane, it was found that the number of light spots had a linear relationship with the concentration of the target DNA (100–1000 copies) (Figure 6b). As the target concentration continually improves, the number of bright spots on the paper will increase, making it difficult to count. When the concentration is less than 100 copies per reaction, there will be no light spots on the paper membrane. Therefore, the linear range is relatively narrow. The inconsistent size of the bright spots may be attributed to the non-uniform pores on the commercialized PES membrane. However, it is a simple and low-cost quantification method because LAMP amplicons can form light spots without preprocessing.

## 4. Conclusions

In this study, we proposed a low-cost and convenient nucleic acid quantification method by performing probe-based LAMP on a paper membrane. The core of digital quantification detection was the separation of the target nucleic acid in different chambers for detection. In this study, we found that the LAMP amplicons can form discrete light spots on the PES membrane. However, the commonly used fluorescence dye-based LAMP had high background signals. By designing and introducing an FIP/Fd probe in the LAMP reaction, the background signal was reduced. After probe-based LAMP, discrete light spots could be observed on the PES membrane. The number of bright spots showed a good correlation with the copy number of the target DNA (100–1000 copies per reaction). The probe-based LAMP for nucleic acid quantification took less than 30 min and the reaction could be performed on a heat plate. Compared with the reported nucleic acid quantification methods, our proposed method was more convenient and could avoid tedious steps. This low-cost and easy-to-operation quantification method was promising for application in point-of-care detection and popularization in low-resourced areas.

## Data Availability

Data are contained within the article and supplementary materials.

## Supplementary Materials

The following supporting information can be downloaded at https://www.mdpi.com/article/10.3390/bios14030139/s1, Table S1: Sequence information used in this paper.
