# Supplementary material for: A Point-of-Care Nucleic Acid Quantification Method by Counting Light Spots Formed by LAMP Amplicons on a Paper Membrane
Source: Biosensors (Basel). 2024 Mar 10;14(3):139. doi: 10.3390/bios14030139 (PMC10967776; doi:10.3390/bios14030139)
Supplement: Supplementary file 1 [file biosensors-14-00139-s001.zip › biosensors-2879822-supplementary.pdf]

Table S1 Sequence information used in this paper.

Name	Sequence (5’ to 3’)
FIP	CCACCAGTAGCCGTCAATGGTGAAGATGATCCAGCGACCGAT
BIP	ACACCAACACGTCGCAAAACGGCGTTCTCGTTCGCCAAAT
F3	ATCGCACCAGCTACTCGA
B3	CGGCGAAGAACGTAATGTCT
LF	TCGTTTTTTGCCCATTCCCA
LB	TTATCCGTCAGCGTTGTGAAGC
F-FIP	FAM-CCACCAGTAGCCGTCAATGGTGAAGATGATCCAGCGACCGAT
Q-Fd	CACCATTGACGGCTACTGGTGG-BHQ1
CrRNA	UAAUUUCUACUGUUGUAGAUAAUGAAACGGAGCUCCACCAG
ssDNA reporters	FAM-CCCCC-BHQ1
